# Evaluation of cytokine analytical performance and optimization of quality control strategies using the six sigma model: a multicenter study

**DOI:** 10.3389/fmed.2026.1904985

**Published:** 2026-07-14

**Authors:** Qian Liu, Tingting Huang, Wei Hu, Yu Lin, Li Mao, Fumeng Yang

**Affiliations:** 1 Department of Laboratory Medicine, Lianyungang Clinical College, Jiangsu University & The Second People's Hospital of Lianyungang, Lianyungang, China; 2 Department of Laboratory Medicine, Affiliated Lianyungang Clinical College of Nantong University, Lianyungang, China; 3 Department of Laboratory Medicine, The Second People's Hospital of Lianyungang Affiliated with Kangda College of Nanjing Medical University, Lianyungang, China; 4 Department of Laboratory Medicine, Lianyungang Clinical College, Bengbu Medical University & The Second People's Hospital of Lianyungang, Lianyungang, China; 5 Department of Laboratory Medicine, Donghai County People's Hospital, Lianyungang, China; 6 Department of Laboratory Medicine, Suqian Hospital of Nanjing Drum Tower Hospital Group, Suqian, China; 7 Department of Laboratory Medicine, Nanping First Hospital, Nanping, China; 8 Department of Laboratory Medicine, The People’s Hospital of Danyang, Zhenjiang, China

**Keywords:** biological variation, cytokines, external quality assessment, quality goal index, six sigma, total allowable error

## Abstract

**Background:**

The six sigma model is widely applied in laboratory quality management. This study used total allowable error (TEa) based on National Center for Clinical Laboratories (NCCL) external quality assessment (EQA) criteria and “desirable” biological variation (BV) specifications as quality goals to evaluate the analytical performance of 12 cytokines in five laboratories and develop individualized quality control (QC) strategies.

**Methods:**

Imprecision and trueness data for 12 cytokine assays were collected from five laboratories and used to calculate sigma metrics. Assay performance was visualized using a normalized sigma method decision chart. The Westgard sigma rules run-size flowchart and quality goal index (QGI) were further applied to design customized QC protocols and identify priority areas for improvement.

**Results:**

Sigma metrics varied by concentration level, with higher-concentration materials generally yielding better sigma performance. For the same analyte, sigma values also differed according to the selected quality goal. Compared with “desirable” BV specifications, NCCL criteria produced lower sigma values for interleukin-6 (IL-6), whereas interferon-*γ* (IFN-γ) and tumor necrosis factor-*α* (TNF-α) showed the opposite trend. These differences were clearly demonstrated by the normalized sigma method decision chart. Based on Westgard sigma rules and QGI, individualized QC strategies and targeted improvement measures were established for assays with sigma values below 6.

**Conclusion:**

The six sigma model provides a practical framework for cytokine assays quality management, supporting targeted QC design and focused analytical performance improvement.

## 1 Introduction

Cytokines are low-molecular-weight soluble polypeptides that serve as important mediators of intercellular communication (1). They are produced by immune cells as well as various tissue-resident cells and participate in the regulation of inflammatory responses and diverse cellular activities (1). In routine clinical laboratory testing, commonly assessed cytokines include interleukin-6 (IL-6), interleukin-8 (IL-8), interleukin-10 (IL-10), interferon-*α* (IFN-*α*), interferon-*γ* (IFN-γ), interleukin-12p70 (IL-12p70), interleukin-17 (IL-17), interleukin-1β (IL-1β), interleukin-2 (IL-2), interleukin-4 (IL-4), interleukin-5 (IL-5), and tumor necrosis factor-α (TNF-α) (2–5). These biomarkers have been increasingly applied in population health screening and in the diagnosis, monitoring, and management of inflammation-related diseases (2–5). Previous studies have demonstrated that cytokines are key regulators of immune homeostasis and are implicated in a broad spectrum of pathological processes. Beyond their physiological roles in immune responses, dysregulated cytokine production contributes to the initiation and progression of various diseases, including inflammatory disorders, infections, malignancies, autoimmune diseases, and coronary artery disease (6–10). Accordingly, accurate *in vitro* quantification of cytokine concentrations is essential for evaluating immune status and providing reliable evidence for clinical decision-making.

As a practical and efficient tool for quality management, the six sigma model was first introduced into clinical laboratory practice by Nevalainen et al. (11) and has subsequently demonstrated substantial value in laboratory performance assessment and quality improvement. The six sigma framework is mainly determined by three parameters: total allowable error (TEa), bias, and coefficient of variation (CV) (12, 13). Bias represents analytical trueness, whereas CV reflects analytical imprecision; together, they describe the overall performance of an analytical system (12, 13). In contrast, TEa is primarily determined by the selected quality specification rather than by the inherent capability of the testing system itself (12, 13). An assay achieving six-sigma performance corresponds to a defect rate of only 3.4 per million results, which is generally regarded as “world-class” quality (12, 13), indicating that the reported results have a high degree of precision and reliability. At the 2014 Milan Strategic Conference, the European Federation of Clinical Chemistry and Laboratory Medicine (EFLM), together with other organizations, proposed three models for defining analytical performance specifications: clinical outcome-based specifications (Model 1), biological variation (BV)-based specifications (Model 2), and state-of-the-art specifications (Model 3) (14, 15). Model 1 is suitable for measurands with well-established clinical utility and clearly defined decision limits; however, only a limited number of analytes currently meet these criteria (14, 15). Model 2 has been widely adopted because BV data are relatively comprehensive and readily available for many analytes (14, 15). Model 3 reflects the best performance currently achievable by available analytical methods and is applicable to analytes that do not play a central role in diagnosis or are not tightly regulated by homeostatic mechanisms. It may also be used as an alternative when Model 1 or Model 2 is not feasible (14, 15). In the present study, we adopted both Model 2, represented by “desirable” BV specifications, and Model 3, represented by the external quality assessment (EQA) criteria of the National Center for Clinical Laboratories (NCCL), as quality goals for cytokine assays. This approach enabled a preliminary evaluation of how different sources of quality goals may influence sigma metrics and their application within the six sigma framework.

Previous studies have shown that the six sigma model has been extensively applied in clinical hematology, coagulation, clinical chemistry, and immunology, providing practical support for continuous quality improvement in laboratory medicine (16–20). Nevertheless, evidence regarding its application to cytokine testing remains scarce. To our knowledge, this is the first study to assess the analytical performance of cytokine assays across five laboratories by integrating the six sigma model with two distinct quality goals: the NCCL EQA criteria and “desirable” BV specifications. In addition, we established individualized quality control (QC) strategies and targeted improvement plans for each laboratory, aiming to provide data-driven evidence to support the clinical implementation and quality management of cytokine testing.

## 2 Materials and methods

### 2.1 Materials

This study was conducted in five laboratories in China, which were coded as Laboratories A–E. The evaluated analytes included IL-6, IL-8, IL-10, IFN-*α*, IFN-*γ*, IL-12p70, IL-17, IL-1β, IL-2, IL-4, IL-5, and TNF-α. Cytokine concentrations were measured using two analytical platforms: the DxFLEX flow cytometer (Beckman Coulter, Brea, CA, USA) and the BD FACSCalibur flow cytometer (BD Biosciences, San Jose, CA, USA). Both platforms used the same 12-plex cytokine assay kit (Qingdao Raisecare Biotechnology Co., Ltd., Qingdao, China; lot No. 250205). A two-level composite cytokine quality control (QC) material (lot No. 2025090100), supplied by Jiangsu Kuole Biotechnology Co., Ltd., was used primarily to evaluate imprecision and trueness.

### 2.2 Methods

Cytokine concentrations were determined using a multiplex microsphere-based flow immunofluorescence assay, following the manufacturer’s instructions. In brief, fluorescent microspheres conjugated with cytokine-specific antibodies and corresponding biotin-labeled detection antibodies capture cytokines in the sample or calibrator, thereby forming sandwich immune complexes. Following incubation with phycoerythrin-labeled streptavidin, the fluorescence signal is detected by flow cytometry, and cytokine concentrations are calculated according to the measured fluorescence intensity.

#### 2.2.1 Calculation of sigma metrics

Sigma metrics for each cytokine were derived from the analytical imprecision (CV) and bias, based on the chosen quality specification. The CV was calculated from two-level internal quality control (IQC) data collected over three consecutive months (September to November 2025) from the five participating laboratories. All laboratories adhered to a uniform IQC protocol: IQC was performed once daily at two concentration levels, with control materials inserted randomly among patient samples. Each laboratory established its own target mean for each analyte from routine testing, and the CV at both IQC levels was subsequently computed (Supplementary Table 1).

Bias, reflecting analytical trueness, was assessed using IQC materials as a surrogate, as the NCCL does not currently offer an EQA program for these 12 cytokines. Briefly, two IQC levels (Level 1 and Level 2) were selected, and each level was measured five consecutive times. The mean of the five measurements from each laboratory was taken as the laboratory-specific measured value. The target value for each analyte was defined as the overall mean of the measured values across all five laboratories. This assigned target value represents the average result of the participating laboratories rather than an absolute true value. However, because all laboratories used the same commercial reagent kit, system-related differences were minimized, thereby enhancing interlaboratory comparability. Bias was calculated as the absolute difference between the laboratory-specific mean and the corresponding target value (Supplementary Table 2).

The sigma metric was calculated using the following general formula (21):


$$\text{Sigma}=\frac{\left[\mathrm{TEa}(\%)-\|\text{Bias}(\%)\|\right]}{\mathrm{CV}(\%)}$$ (1)

Note to Equation (1): TEa denotes the allowable total error, representing the quality specification selected by each laboratory. In this study, two quality goals were applied: the NCCL EQA standard (22) and the “desirable” BV specification (23), and the resultant sigma values were compared. Of the 12 cytokines, BV-derived TEa values are currently available only for IL-6, IFN-*γ*, and TNF-*α*; data for the remaining analytes are not yet accessible.

Bias was calculated as:


$$\text{Valu}e_{\text{average}}=\frac{\text{Valu}e_{1}+\text{Valu}e_{2}+\text{Valu}e_{3}+\text{Valu}e_{4}+\text{Valu}e_{5}}{5}$$ (2)

$$\text{Bias}=\frac{|\text{Valu}e_{\text{average}}-\text{Valu}e_{\text{target}}|}{\text{Valu}e_{\text{target}}}\times 100\%$$

Note to Equation (2): Third-party quality control materials are employed as evaluation substances for the surrogate assessment of correctness. Each participating laboratory performs measurements on two concentration levels of these control materials in accordance with the above formula.

#### 2.2.2 Normalized sigma method decision charts for cytokines

Normalized sigma method decision charts were constructed using the Laboratory Medicine Information Network platform.^1^ Analytical performance data for the cytokine assays at two concentration levels (Level 1 and Level 2) from the five participating laboratories were mapped onto the charts. In each chart, Bias/TEa (%) is displayed on the y-axis, whereas CV/TEa (%) is displayed on the x-axis. The decision region is separated by five straight lines into six performance zones, each corresponding to a specific sigma level. From the lower-left to the upper-right region, the zones indicate sigma ≥ 6, 6 > sigma ≥ 5, 5 > sigma ≥ 4, 4 > sigma ≥ 3, 3 > sigma ≥ 2, and sigma < 2, respectively (24).

#### 2.2.3 Design of individualized QC strategies and improvement measures

**Figure 1:**
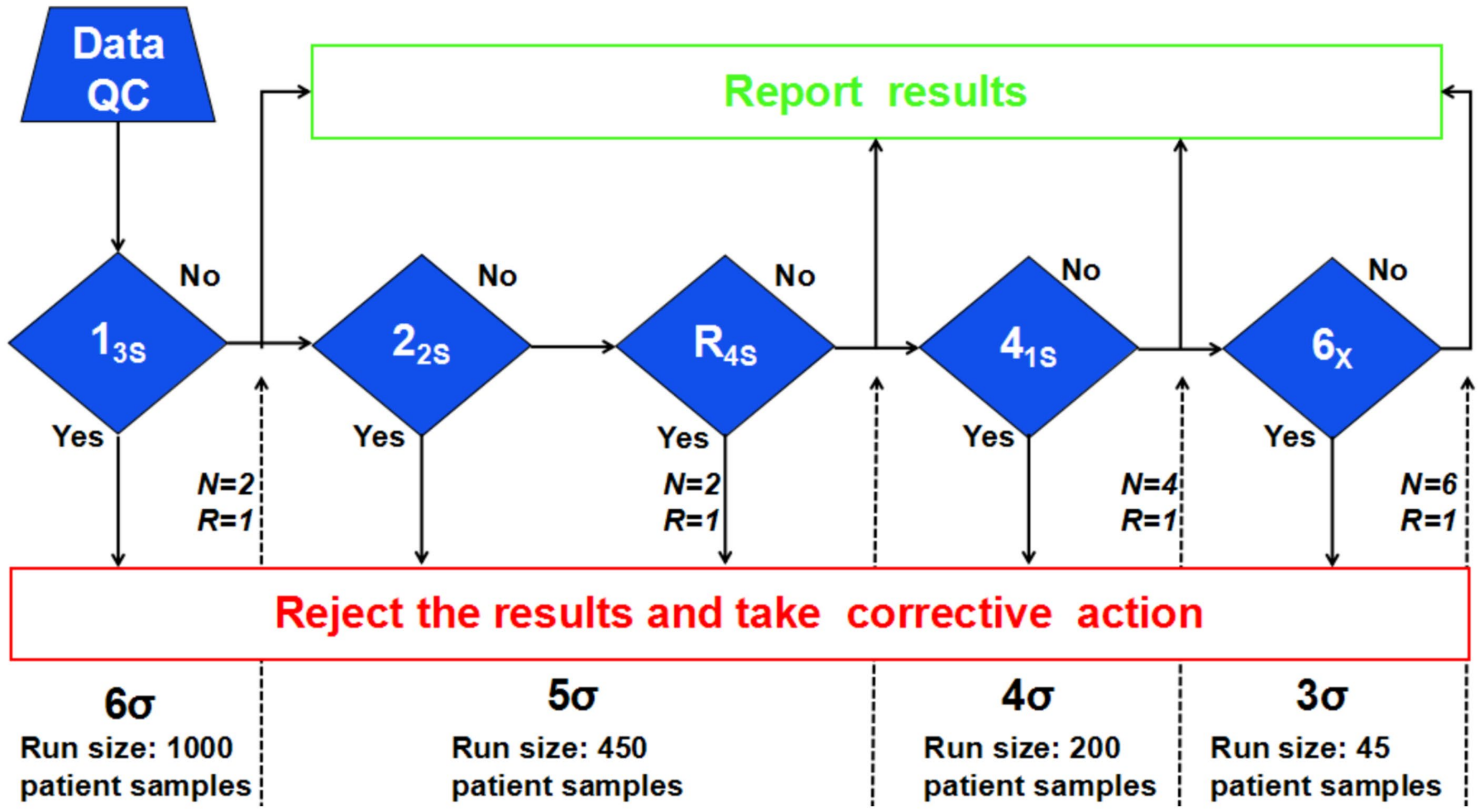
Flow chart of Westgard sigma rules with run sizes (cited from website http://www.clinet.com.cn/sigmapv/#sgm4). Sigma metrics were calculated as Sigma = [TEa (%) − |bias (%)|]/CV (%). For each assay, the sigma value was first derived using this formula. The corresponding QC rules, number of QC samples (N), and analytical run size (batch length) were then determined according to the sigma scale shown at the bottom of the flowchart. A result of “Yes” indicates that a QC rule has been violated; in that case, the analytical run is rejected and corrective action is initiated. A result of “No” indicates that no QC rule violation occurred, and the results can be accepted and reported.

Individualized QC strategies for cytokine assays in the five laboratories were established according to the Westgard sigma rules run-size flowchart (Figure 1) and the sigma metric calculated for each analyte. For analytes with sigma values <6, the quality goal index (QGI) was additionally determined to identify the major contributors to suboptimal analytical performance. QGI was calculated using the following formula:


$$\mathrm{QGI}=\frac{|\text{Bias}(\%)|}{1.5\times \mathrm{CV}(\%)}$$ (3)

Note to Equation (3): A QGI < 0.8 suggests that improvement should primarily target imprecision; a QGI > 1.2 indicates that trueness should be prioritized; and a QGI between 0.8 and 1.2 implies that both imprecision and trueness require simultaneous improvement (25).

## 3 Results

### 3.1 Sigma values of cytokines using the EQA standard of NCCL as the quality goal

The EQA criteria of the NCCL represent state-of-the-art analytical performance specifications based on current clinical laboratory practice (Model 3). In this study, these criteria were adopted as the quality goal for calculating sigma metrics for cytokine assays across the five laboratories. The results indicated that sigma metrics for the same analyte varied across concentration levels, with higher concentrations generally corresponding to higher sigma values. For instance, in Laboratory A, the sigma metric for IL-8 was 3.97 at QC Level 1 and increased to 4.43 at QC Level 2. Comparable trends were observed for other analytes (Table 1).

**Table 1:** Sigma metrics for cytokines in five laboratories using NCCL’s EQA criteria as the quality goal.

Cytokines	TEa (EQA standard NCCL)	Sigma metrics of Lab A		Sigma metrics of Lab B		Sigma metrics of Lab C		Sigma metrics of Lab D		Sigma metrics of Lab E	
		Level 1	Level 2	Level 1	Level 2	Level 1	Level 2	Level 1	Level 2	Level 1	Level 2
IL-6	30.00%	4.08	4.57	5.53	5.58	4.40	4.89	4.63	4.98	5.13	5.90
IL-8	30.00%	3.97	4.43	5.89	6.81	4.71	5.53	4.07	5.16	5.79	5.86
IL-10	30.00%	4.46	5.80	4.88	5.97	4.32	4.92	5.05	5.50	4.67	5.56
IFN-α*	30.00%	5.16	5.34	5.31	5.52	4.56	4.81	6.08	7.42	4.22	4.67
IFN-γ*	30.00%	4.05	5.23	4.78	5.68	4.61	5.56	5.09	5.93	4.17	4.75
IL-12p70*	30.00%	5.71	5.92	5.38	5.80	4.39	5.03	5.10	5.51	4.61	4.85
IL-17*	30.00%	4.90	5.13	5.43	6.12	4.22	5.09	5.48	5.85	4.39	4.92
IL-1β*	30.00%	4.20	4.49	4.70	5.52	4.97	5.83	5.31	6.78	4.17	4.63
IL-2*	30.00%	4.04	4.60	5.37	5.88	4.66	4.86	4.52	5.01	4.59	5.44
IL-4*	30.00%	3.62	4.44	4.84	5.12	4.21	4.80	4.90	5.41	4.86	5.22
IL-5*	30.00%	4.78	5.01	5.35	6.43	5.02	5.23	5.11	5.61	5.80	6.25
TNF-α*	30.00%	5.35	5.42	5.63	6.26	4.83	5.35	5.70	7.19	4.80	5.81

TEa, total allowable error; EQA, external quality assessment; NCCL, National Center for Clinical Laboratories; IL-6, interleukin-6; IL-8, interleukin-8; IL-10, interleukin-10; IFN-α, interferon-α; IFN-γ, interferon-gamma; IL-12p70, interleukin-12p70; IL-17, interleukin-17; IL-1β, interleukin-1β; IL-2, interleukin-2; IL-4, interleukin-4; IL-5, interleukin-5; TNF-α, tumor necrosis factor-α.

*Indicates that the NCCL has not published TEa for this analyte; TEa values for IL-6, IL-8, and IL-10 were used as reference values.

**Figure 2:**
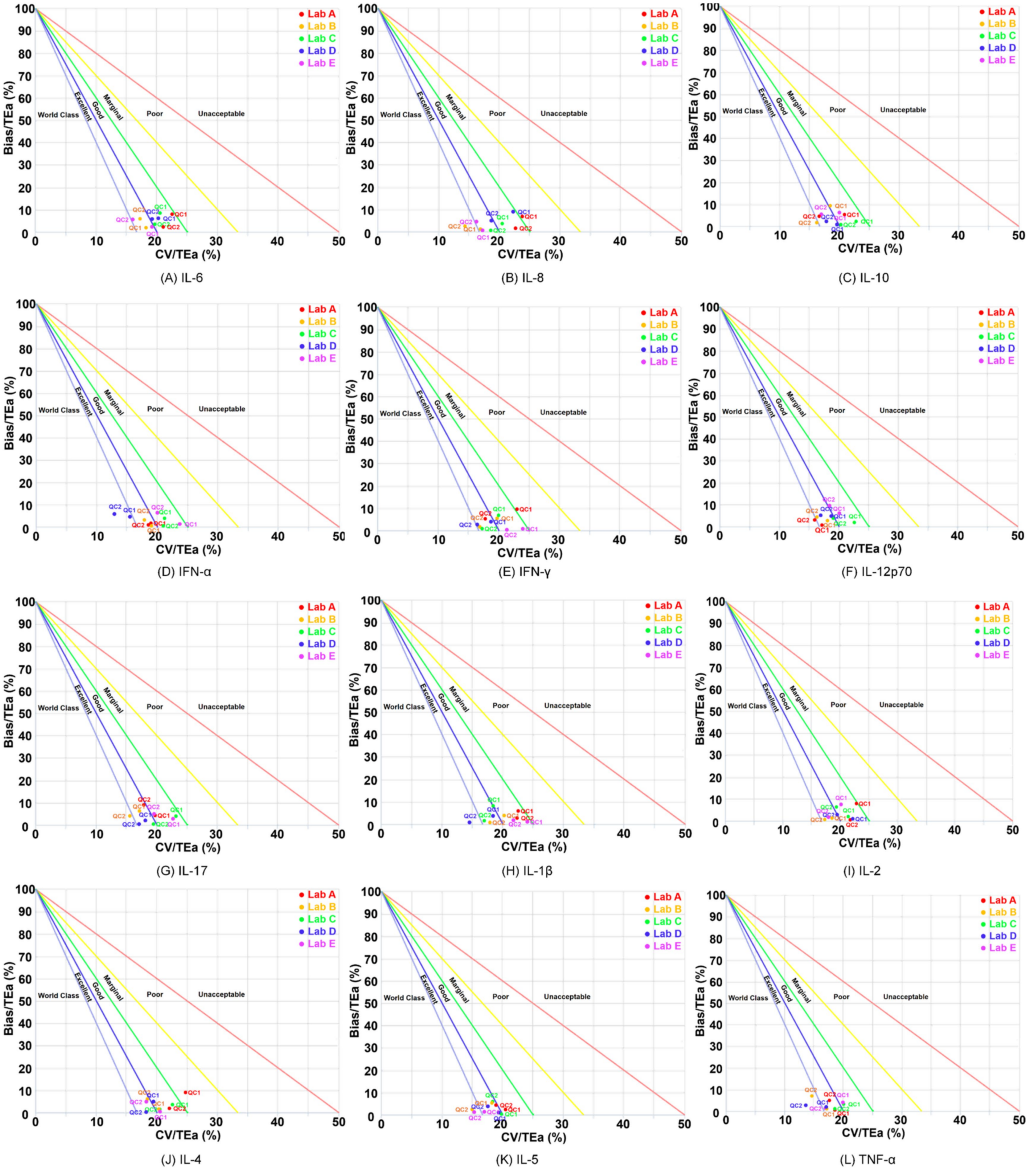
Normalized sigma method decision charts for cytokines using the EQA standard NCCL as the quality goal **(A–L)**. As shown in the figure, the slopes of the five straight lines represent negative sigma values. Thus, when an assay falls on one of these lines, the magnitude of the negative slope corresponds to the sigma metric of the assay. Imprecision is represented on the x-axis (CV/TEa, %), and trueness is represented on the y-axis (Bias/TEa, %).

Overall, the sigma metrics of most cytokines across the five laboratories were distributed between 4 and 6. Only a small number of analytes reached a sigma level of 6, including IL-8 (Level 2), IL-17 (Level 2), IL-5 (Level 2), and TNF-*α* (Level 2) in Laboratory B; IFN-α (Levels 1 and 2), IL-1β (Level 2), and TNF-*α* (Level 2) in Laboratory D; and IL-5 (Level 2) in Laboratory E. These findings were visually summarized using normalized sigma method decision charts (Figure 2).

### 3.2 Sigma values of cytokines using the “desirable” BV as quality goal

**Figure 3:**
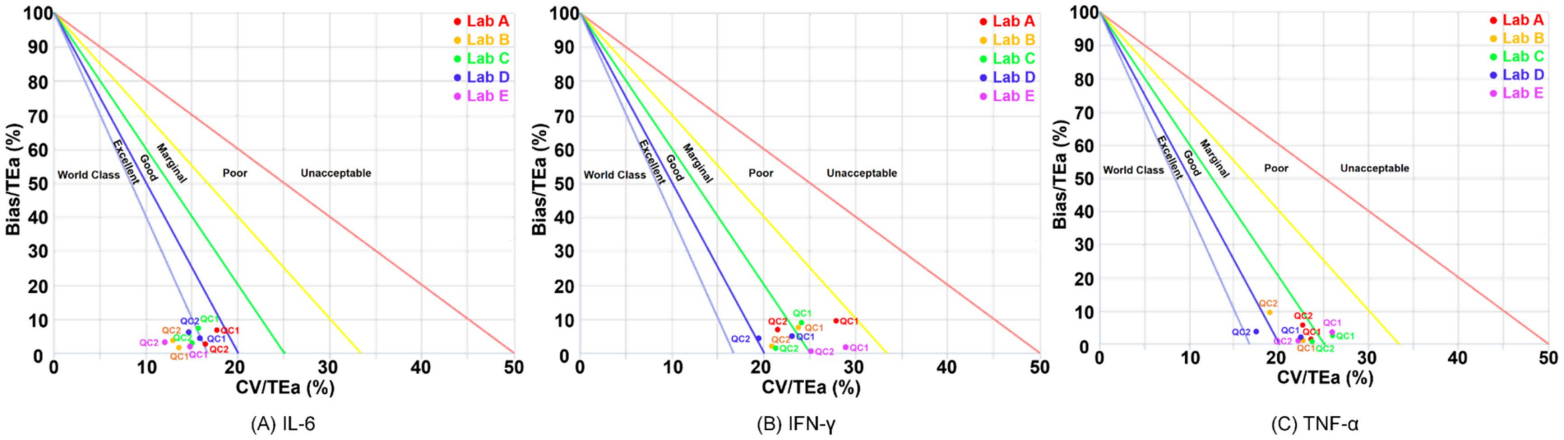
Normalized sigma method decision charts for cytokines using “desirable” BV as the quality goal **(A–C)**. As shown in the figure, the slopes of the five straight lines represent negative sigma values. Thus, when an assay falls on one of these lines, the magnitude of the negative slope corresponds to the sigma metric of the assay. Imprecision is represented on the x-axis (CV/TEa, %), and trueness is represented on the y-axis (Bias/TEa, %).

Because TEa values from the BV database were available only for IL-6, IFN-*γ*, and TNF-*α*, BV-based sigma metrics were calculated exclusively for these three cytokines across the five laboratories. The sigma metrics for the three analytes ranged from 3.32 to 7.77. Among them, IL-6 demonstrated the most favorable analytical performance, with 70% of measurements attaining the six-sigma level. In contrast, IFN-γ and TNF-α failed to reach six-sigma performance under the BV-based quality goal (Table 2). The normalized sigma method decision charts further provided a visual representation of the analytical performance of these three cytokines across the five laboratories (Figure 3).

**Table 2:** Sigma metrics of cytokines in five laboratories using “desirable” BV as the quality goal.

Cytokines*	TEa (“desirable” BV)	Sigma metrics of Lab A		Sigma metrics of Lab B		Sigma metrics of Lab C		Sigma metrics of Lab D		Sigma metrics of Lab E	
		Level 1	Level 2	Level 1	Level 2	Level 1	Level 2	Level 1	Level 2	Level 1	Level 2
IL-6	39.00	5.40	5.98	7.22	7.35	5.84	6.42	6.09	6.55	6.71	7.77
IFN-γ	25.00	3.32	4.31	3.94	4.72	3.79	4.62	4.20	4.91	3.47	3.96
TNF-α	23.20	4.13	4.12	4.35	4.73	3.71	4.13	4.38	5.51	3.69	4.48

TEa, total allowable error; BV, biological variation; IL-6, interleukin-6; IFN-γ, interferon-γ; TNF-α, tumor necrosis factor-α.

*Indicates that the biological variation database currently provides TEa values only for IL-6, IFN-γ, and TNF-α; TEa data for the other cytokines are not yet available, and therefore no corresponding analysis was performed.

### 3.3 Comparison of sigma values for cytokines derived from different quality goals

**Figure 4:**
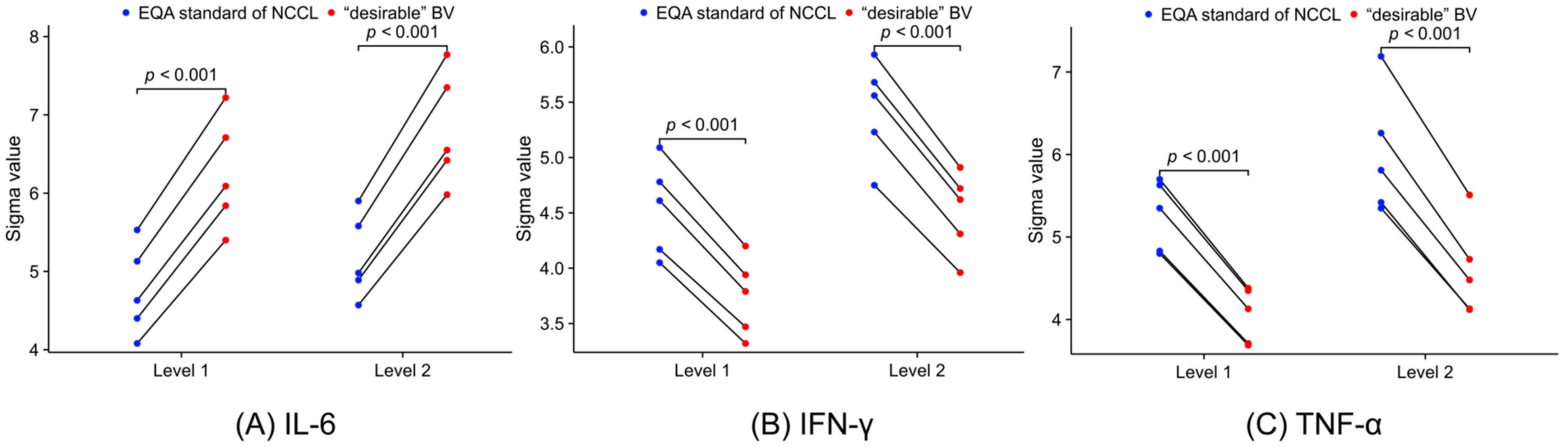
Comparison of cytokine sigma metrics across distinct quality goal sources **(A–C)**. As shown in the figure, the x-axis represents the QC material level, and the y-axis represents the sigma metric. Paired t tests were performed separately for the sigma metrics of three cytokines (IL-6, IFN-*γ*, and TNF-*α*) across the five laboratories, and a *p* value < 0.05 was considered statistically significant.

Our findings revealed that sigma metrics varied considerably depending on the chosen quality specification. When the NCCL EQA criteria were applied, IFN-*γ* and TNF-*α* yielded substantially higher sigma values than those calculated using the “desirable” BV-based goals, whereas an opposite pattern was observed for IL-6 (Figure 4).

### 3.4 Individualized QC strategy formulation for cytokines

Using the NCCL EQA criteria as the performance benchmark, we established individualized QC strategies for each cytokine based on Westgard sigma rules run-size flowchart and the analyte-specific sigma metrics (Table 3). For example, IL-6 in Laboratory A showed a sigma value between 4 and 5; therefore, a multirule QC procedure consisting of 1_3s_/2_2s_/R_4s_/4_1s_ was recommended, with *N* = 4 and a maximum run size of 200 patient samples. In contrast, IFN-*α* in Laboratory D achieved six-sigma performance, for which a simpler 1_3s_ single-rule QC procedure with *N* = 2 and a maximum run size of 1,000 patient samples was considered appropriate. When different sigma values were obtained at distinct concentration levels for the same analyte, the QC strategy was defined based on the lower sigma metric. For instance, IL-8 in Laboratory A had sigma values of 3.97 at QC Level 1 and 4.43 at QC Level 2. Accordingly, the 1_3s_/2_2s_/R_4s_/4_1s_/6_X_ rule set was applied, with *N* = 6 and a maximum run size of 45 patient samples.

**Table 3:** Individualized internal quality control strategies for cytokines across five laboratories.

Cytokines	Internal quality control strategies				
	Lab A	Lab B	Lab C	Lab D	Lab E
IL-6	1_3s_/2_2s_/R_4s_/4_1s_ with *N* = 4 and R = 200	1_3s_/2_2s_/R_4s_ with *N* = 2 and R = 450	1_3s_/2_2s_/R_4s_/4_1s_ with *N* = 4 and R = 200	1_3s_/2_2s_/R_4s_/4_1s_ with *N* = 4 and R = 200	1_3s_/2_2s_/R_4s_ with *N* = 2 and R = 450
IL-8	1_3s_/2_2s_/R_4s_/4_1s_/6_X_ with *N* = 6 and R = 45	1_3s_/2_2s_/R_4s_ with *N* = 2 and R = 450	1_3s_/2_2s_/R_4s_/4_1s_ with *N* = 4 and R = 200	1_3s_/2_2s_/R_4s_/4_1s_ with *N* = 4 and R = 200	1_3s_/2_2s_/R_4s_ with *N* = 2 and R = 450
IL-10	1_3s_/2_2s_/R_4s_/4_1s_ with *N* = 4 and R = 200	1_3s_/2_2s_/R_4s_/4_1s_ with *N* = 4 and R = 200	1_3s_/2_2s_/R_4s_/4_1s_ with *N* = 4 and R = 200	1_3s_/2_2s_/R_4s_ with *N* = 2 and R = 450	1_3s_/2_2s_/R_4s_/4_1s_ with *N* = 4 and R = 200
IFN-α	1_3s_/2_2s_/R_4s_ with *N* = 2 and R = 450	1_3s_/2_2s_/R_4s_ with *N* = 2 and R = 450	1_3s_/2_2s_/R_4s_/4_1s_ with *N* = 4 and R = 200	1_3s_ with *N* = 2 and R = 1,000	1_3s_/2_2s_/R_4s_/4_1s_ with *N* = 4 and R = 200
IFN-γ	1_3s_/2_2s_/R_4s_/4_1s_ with *N* = 4 and R = 200	1_3s_/2_2s_/R_4s_/4_1s_ with *N* = 4 and R = 200	1_3s_/2_2s_/R_4s_/4_1s_ with *N* = 4 and R = 200	1_3s_/2_2s_/R_4s_ with *N* = 2 and R = 450	1_3s_/2_2s_/R_4s_/4_1s_ with *N* = 4 and R = 200
IL-12p70	1_3s_/2_2s_/R_4s_ with *N* = 2 and R = 450	1_3s_/2_2s_/R_4s_ with *N* = 2 and R = 450	1_3s_/2_2s_/R_4s_/4_1s_ with *N* = 4 and R = 200	1_3s_/2_2s_/R_4s_ with *N* = 2 and R = 450	1_3s_/2_2s_/R_4s_/4_1s_ with *N* = 4 and R = 200
IL-17	1_3s_/2_2s_/R_4s_/4_1s_ with *N* = 4 and R = 200	1_3s_/2_2s_/R_4s_ with *N* = 2 and R = 450	1_3s_/2_2s_/R_4s_/4_1s_ with *N* = 4 and R = 200	1_3s_/2_2s_/R_4s_ with *N* = 2 and R = 450	1_3s_/2_2s_/R_4s_/4_1s_ with *N* = 4 and R = 200
IL-1β	1_3s_/2_2s_/R_4s_/4_1s_ with *N* = 4 and R = 200	1_3s_/2_2s_/R_4s_/4_1s_ with *N* = 4 and R = 200	1_3s_/2_2s_/R_4s_/4_1s_ with *N* = 4 and R = 200	1_3s_/2_2s_/R_4s_ with *N* = 2 and R = 450	1_3s_/2_2s_/R_4s_/4_1s_ with *N* = 4 and R = 200
IL-2	1_3s_/2_2s_/R_4s_/4_1s_ with *N* = 4 and R = 200	1_3s_/2_2s_/R_4s_ with *N* = 2 and R = 450	1_3s_/2_2s_/R_4s_/4_1s_ with *N* = 4 and R = 200	1_3s_/2_2s_/R_4s_/4_1s_ with *N* = 4 and R = 200	1_3s_/2_2s_/R_4s_/4_1s_ with *N* = 4 and R = 200
IL-4	1_3s_/2_2s_/R_4s_/4_1s_/6_X_ with *N* = 6 and R = 45	1_3s_/2_2s_/R_4s_/4_1s_ with *N* = 4 and R = 200	1_3s_/2_2s_/R_4s_/4_1s_ with *N* = 4 and R = 200	1_3s_/2_2s_/R_4s_/4_1s_ with *N* = 4 and R = 200	1_3s_/2_2s_/R_4s_/4_1s_ with *N* = 4 and R = 200
IL-5	1_3s_/2_2s_/R_4s_/4_1s_ with *N* = 4 and R = 200	1_3s_/2_2s_/R_4s_ with *N* = 2 and R = 450	1_3s_/2_2s_/R_4s_ with *N* = 2 and R = 450	1_3s_/2_2s_/R_4s_ with *N* = 2 and R = 450	1_3s_/2_2s_/R_4s_ with *N* = 2 and R = 450
TNF-α	1_3s_/2_2s_/R_4s_ with *N* = 2 and R = 450	1_3s_/2_2s_/R_4s_ with *N* = 2 and R = 450	1_3s_/2_2s_/R_4s_/4_1s_ with *N* = 4 and R = 200	1_3s_/2_2s_/R_4s_ with *N* = 2 and R = 450	1_3s_/2_2s_/R_4s_/4_1s_ with *N* = 4 and R = 200

IL-6, interleukin-6; IL-8, interleukin-8; IL-10, interleukin-10; IFN-α, interferon-α; IFN-γ, interferon-γ; IL-12p70, interleukin-12p70; IL-17, interleukin-17; IL-1β, interleukin-1 beta; IL-2, interleukin-2; IL-4, interleukin-4; IL-5, interleukin-5; TNF-α, tumor necrosis factor-α. *N* = number of QC measurements; R = number of patient samples tested between QC events. These IQC strategies were newly implemented after review of the sigma metrics.

### 3.5 QGI and improvement strategies for cytokines

For analytes with sigma <6, the QGI was calculated to identify the predominant cause of suboptimal performance. All cytokines showed QGI values below 0.8, indicating that imprecision was the major limiting factor. Accordingly, we implemented measures targeting five key aspects—personnel, equipment, reagents, methodology, and environment—as follows: (1) Personnel: enhanced training and competency assessments were conducted across pre-analytical, analytical, and post-analytical phases, with all procedures strictly following standard operating protocols to ensure inter-operator consistency; (2) Equipment: maintenance was performed per the manufacturer’s schedules (daily, weekly, monthly), with additional checks after repairs; (3) Reagents and consumables: stored and used according to specifications, with strict prohibition of mixing different lots; (4) Methodology: analytical performance of each kit was verified to confirm that claimed specifications (especially imprecision) met testing requirements, prioritizing methods with higher reliability for accurate and stable results; (5) Environment: workplace temperature and humidity were closely monitored to comply with instrument operational criteria (Table 4).

**Table 4:** Quality goal index and improvement strategies for cytokines exhibiting sigma metrics below 6.

Cytokines	QGI of Lab A			QGI of Lab B			QGI of Lab C			QGI of Lab D			QGI of Lab E		
	Level 1	Level 2	Improvement measures	Level 1	Level 2	Corrective measures	Level 1	Level 2	Corrective measures	Level 1	Level 2	Corrective measures	Level 1	Level 2	Corrective measures
IL-6	0.22	0.09	Imprecision	0.08	0.21	Imprecision	0.26	0.13	Imprecision	0.16	0.17	Imprecision	0.09	0.22	Imprecision
IL-8	0.21	0.04	Imprecision	0.03	-	Imprecision	0.07	0.01	Imprecision	0.29	0.17	Imprecision	0.03	0.17	Imprecision
IL-10	0.16	0.13	Imprecision	0.35	0.05	Imprecision	0.05	0.00	Imprecision	0.01	0.07	Imprecision	0.22	0.16	Imprecision
IFN-α	0.07	0.05	Imprecision	0.01	0.11	Imprecision	0.11	0.01	Imprecision	-	-	-	0.04	0.21	Imprecision
IFN-γ	0.22	0.19	Imprecision	0.19	0.05	Imprecision	0.22	0.04	Imprecision	0.14	0.11	Imprecision	0.03	0.00	Imprecision
IL-12p70	0.05	0.12	Imprecision	0.07	0.17	Imprecision	0.05	0.10	Imprecision	0.12	0.20	Imprecision	0.18	0.33	Imprecision
IL-17	0.13	0.34	Imprecision	0.26	-	Imprecision	0.08	0.03	Imprecision	0.09	0.01	Imprecision	0.07	0.14	Imprecision
IL-1β	0.19	0.06	Imprecision	0.10	0.03	Imprecision	0.27	0.05	Imprecision	0.13	-	Imprecision	0.05	0.04	Imprecision
IL-2	0.25	0.02	Imprecision	0.04	0.00	Imprecision	0.03	0.19	Imprecision	0.02	0.08	Imprecision	0.24	0.09	Imprecision
IL-4	0.26	0.05	Imprecision	0.05	0.17	Imprecision	0.13	0.04	Imprecision	0.12	0.03	Imprecision	0.02	0.13	Imprecision
IL-5	0.08	0.12	Imprecision	0.16	-	Imprecision	0.00	0.18	Imprecision	0.02	0.12	Imprecision	0.05	-	Imprecision
TNF-α	0.02	0.20	Imprecision	0.03	-	Imprecision	0.07	0.00	Imprecision	0.07	-	Imprecision	0.09	0.03	Imprecision

QGI, quality goal index; IL-6, interleukin-6; IL-8, interleukin-8; IL-10, interleukin-10; IFN-α, interferon-α; IFN-γ, interferon-γ; IL-12p70, interleukin-12p70; IL-17, interleukin-17; IL-1β, interleukin-1 beta; IL-2, interleukin-2; IL-4, interleukin-4; IL-5, interleukin-5; TNF-α, tumor necrosis factor-α.

“-” indicates that the sigma metric for this analyte reached 6; therefore, no corrective action was required.

## 4 Discussion

As a key approach for lean quality management in clinical laboratories, the six sigma model has been widely recognized and increasingly implemented in laboratory practice (26, 27). To our knowledge, this is the first multicenter study to apply the six sigma model to the performance evaluation of cytokine assays. In accordance with the consensus established at the Milan Strategic Conference (14), we adopted Model 2, represented by “desirable” BV specifications, and Model 3, represented by the NCCL EQA criteria, to systematically assess the analytical performance of 12 cytokines.

Our results demonstrated that sigma metrics for the same cytokine varied across concentration levels, with higher concentrations generally corresponding to higher sigma values. This finding suggests that analyte concentration should be considered an important factor when applying the six sigma model to evaluate cytokine assay performance. We also found that, even under the same quality goal, the sigma performance of a given analyte differed among laboratories. These observations are consistent with previous studies in other areas of laboratory medicine. Tumrani et al. (28) evaluated 20 clinical chemistry analytes using the six sigma model and reported pronounced concentration-dependent differences in sigma values for the same analyte. For instance, glucose showed sigma values of 3.79 and 11.18 at Levels 1 and 2, respectively. Similarly, Rajagopal et al. (29) applied the six sigma model to five core hematology parameters, including hemoglobin, white blood cells, red blood cells, hematocrit, and platelets, and observed substantial variation in sigma values for the same analyte across three concentration levels. When “desirable” BV was used as the quality goal, the sigma values for white blood cells at the three concentrations were 6.131, 4.847, and 5.542, respectively. Üge et al. (30) further assessed 17 biochemical analytes within the six sigma framework and also found clear concentration-related differences. For example, when the Clinical Laboratory Improvement Amendments 1988 (CLIA’88) criteria were adopted as the quality goal, albumin showed sigma values of 1.72 and 2.15 at Levels 1 and 2, respectively. Although these studies were conducted in single-center settings, their findings are highly consistent with ours, supporting the need for concentration-stratified evaluation when applying the six sigma model. This is particularly relevant for analyte concentrations close to clinical decision limits, where inaccurate performance assessment may directly influence clinical interpretation and decision-making.

In the present study, two sources of TEa were used to assess the sigma performance of cytokine assays. The NCCL EQA criteria were adopted as the primary quality goal, while “desirable” BV specifications were used for supplementary analysis. This design allowed us to explore how different quality goals affect the evaluation of analytical performance in cytokine testing. When the NCCL EQA criteria were applied, only a limited number of cytokines achieved six-sigma performance. Most analytes showed sigma values between 4 and 6, and the same cytokine generally exhibited higher sigma values at higher concentration levels. In contrast, when “desirable” BV specifications were used, 70% of IL-6 measurements across the five laboratories reached the six-sigma level, and IL-6 demonstrated substantially higher sigma values than those calculated using the NCCL EQA criteria. However, IFN-*γ* and TNF-*α* showed the opposite pattern. This discrepancy may be largely attributed to the fact that, except for IL-6, BV-derived quality goals were more stringent than the NCCL EQA criteria. Furthermore, when laboratories adopted the “desirable” BV as the quality specification, IFN-*γ* showed lower sigma levels than both IL-6 and TNF-*α*. Several factors may contribute to this finding: (1) the physiological concentration of IFN-γ in serum/plasma is extremely low, coupled with a relatively large within-subject biological variation, which renders the BV-based quality goal highly demanding; (2) compared with IL-6 and TNF-α, the IFN-γ displays higher between-run imprecision (CV%), possibly related to antibody-pair affinity and matrix effects. Rajagopal et al. (29) applied four different quality specifications to calculate sigma metrics for hematology parameters and observed substantial variation according to the source of the quality goal. In their study, “desirable” BV specifications provided stricter TEa limits than those derived from CLIA and the Royal College of Pathologists of Australasia (RCPA). Hkimi et al. (31) evaluated 22 biochemical and immunological analytes using sigma metrics based on two quality goals, namely the Ricos “desirable” criteria and the EFLM BV database. They found marked differences in sigma distributions between these two BV-based standards, with the EFLM database imposing more stringent quality requirements. Similarly, Othmani et al. (32) compared three TEa sources for 14 routine chemistry analytes and reported considerable discrepancies in sigma metrics across different standards. They emphasized the lack of harmonization among existing TEa specifications and suggested the development of a standardized TEa framework that is both clinically meaningful and practically achievable. Ramachandra et al. (33) also reported that different TEa databases may result in substantial variation in sigma metrics and recommended that laboratories select the most appropriate specification according to their own testing conditions. Their study favored the EFLM database because of its broad coverage and stringent criteria. Taken together, these studies and our findings indicate that the source of TEa has a major impact on sigma-based performance evaluation. Therefore, laboratories should carefully select quality goals to ensure accurate, reliable, and clinically relevant assessment of assay performance. In addition, we used normalized sigma method decision charts to further visualize the analytical performance of each cytokine, allowing the sigma level of each analyte to be interpreted more intuitively.

Westgard and colleagues (34–36) have made longstanding contributions to laboratory QC and quality management. By combining statistical models with risk-management principles, they have promoted the development and application of Westgard multirules, Westgard sigma rules, and the run-size-based Westgard sigma rules flowchart in clinical laboratory practice. Before the present study, all five laboratories applied the same QC strategy for cytokine assays, namely the 1_3s_/2_2s_/R_4s_ multirule procedure, without defining a specific allowable batch size during routine testing. In this study, individualized QC strategies were developed according to the Westgard sigma rules run-size flowchart and the sigma performance of each cytokine. Yi et al. (37) conducted a single-center study in which sigma metrics were used to evaluate 41 biochemical analytes in a clinical chemistry laboratory and to optimize IQC protocols according to sigma levels. They concluded that individualized QC strategies can help balance analytical risk and QC cost. Analytes with high sigma performance may be monitored using simpler rules and longer run sizes, thereby reducing QC burden, whereas analytes with lower sigma performance require multirule procedures and shorter run sizes to decrease the probability of reporting erroneous results. Similarly, Choi et al. (38) demonstrated that sigma-based individualized QC rules improved both efficiency and quality. By assigning appropriate Westgard rules to different analytes, including less stringent rules for selected high-sigma analytes, they reduced unnecessary QC repeats and testing delays without compromising proficiency-testing performance; in some cases, performance even improved. These findings are consistent with our results. In the present study, different QC rules and testing frequencies were assigned to each analyte in each laboratory based on its sigma performance. Additional IQC measurements were scheduled before the maximum allowable run length was reached, thereby helping maintain analytical reliability throughout patient testing. Importantly, our study established analyte-specific IQC protocols according to actual analytical performance rather than applying a uniform QC model to all assays. This stratified quality-management strategy may improve analytical reliability while optimizing resource utilization by reducing unnecessary consumption of IQC materials and related reagents without weakening quality requirements. For analytes with sigma values below 6, QGI was calculated to identify the main contributors to unsatisfactory analytical performance and to guide corrective actions. Tumrani et al. (28) calculated QGI for clinical chemistry analytes with sigma values ≤3 and proposed priority directions for improvement. Yi et al. (37) also assessed QGI for analytes with sigma values below 3 and recommended targeted interventions, including more frequent equipment maintenance, closer reagent monitoring, enhanced staff training, selection of more appropriate reagents and liquid QC materials, and periodic reassessment of sigma metrics. Gadde et al. (39) similarly calculated QGI for analytes with poor analytical performance and adjusted QC strategies on the basis of QGI results and root-cause analysis. Collectively, previous studies and our findings indicate that QGI can rapidly identify the predominant source of poor performance and provide a practical basis for targeted corrective action. Although this was a multicenter study involving five laboratories, all participating laboratories showed a common limitation in cytokine testing, namely insufficient precision. Therefore, improvement efforts should prioritize the reduction of analytical imprecision. To support this objective, we proposed a systematic improvement plan covering five domains: personnel, equipment, reagents and consumables, methodology, and the testing environment.

Several limitations of this study should be noted. First, TEa is a critical component of laboratory quality goals and directly determines the calculation of sigma metrics. Although two quality goals were applied in this study, BV-based specifications were not available for all cytokines. Consequently, the BV-based evaluation was incomplete and could only be conducted for analytes with available BV data. Second, trueness was evaluated using a surrogate approach. Because certified reference materials and reference measurement procedures are currently unavailable, and because the NCCL has not yet established an external quality assessment program for these 12 cytokines, an alternative strategy had to be adopted to assess trueness. Third, although this multicenter study analyzed the causes of suboptimal performance across five laboratories and proposed targeted improvement measures, the actual implementation of these interventions and evaluation of their effectiveness require continued monitoring and feedback from the participating laboratories. Therefore, further validation is needed before these findings can be generalized to broader clinical laboratory settings.

## 5 Conclusion

In conclusion, this is the first study to assess the analytical performance of cytokine assays across five laboratories by integrating the six sigma model with two sources of quality goals: the NCCL EQA criteria and “desirable” BV specifications. On the basis of these assessments, individualized QC strategies and targeted improvement plans were established for each laboratory. Our findings suggest that the six sigma model is a practical and effective approach for laboratory quality management. Its application may help laboratories optimize cytokine assay performance and provide reliable analytical support for the clinical use of cytokine testing, although prospective validation is still required.

## Data Availability

The original contributions presented in the study are included in the article/Supplementary material, further inquiries can be directed to the corresponding author.

## Footnotes

1
www.clinet.com.cn

## Glossary

IL-6: Interleukin-6
IL-8: Interleukin-8
IL-10: Interleukin-10
IFN-*α*: Interferon-α
IFN-*γ*: Interferon-γ
IL-12p70: Interleukin-12p70
IL-17: Interleukin-17
IL-1β: Interleukin-1β
IL-2: Interleukin-2
IL-4: Interleukin-4
IL-5: Interleukin-5
TNF-α: tumor necrosis factor-α
TEa: Total allowable error
CV: Coefficient of variation
EFLM: European Federation of Clinical Chemistry and Laboratory Medicine
BV: Biological variation
EQA: External quality assessment
NCCL: National Center for Clinical Laboratories
QC: Quality control
IQC: Internal quality control
QGI: Quality goal index
CLIA’88: Clinical Laboratory Improvement Amendments 1988
RCPA: Royal College of Pathologists of Australasia.
