# Supplementary material for: Evaluation of cytokine analytical performance and optimization of quality control strategies using the six sigma model: a multicenter study
Source: Front Med (Lausanne). 2026 Jul 14;13:1904985. doi: 10.3389/fmed.2026.1904985 (PMC13408381; doi:10.3389/fmed.2026.1904985)
Supplement: Supplementary file 1 [file Supplementary_file_1.DOC]

**Supplementary Table 1.** Cytokine CVs across five laboratories under two quality control levels (%)

| Cytokines | CV of Lab A | | CV of Lab B | | CV of Lab C | | CV of Lab D | | CV of Lab E | |
| --- | --- | --- | --- | --- | --- | --- | --- | --- | --- | --- |
|  | Level 1 | Level 2 | Level 1 | Level 2 | Level 1 | Level 2 | Level 1 | Level 2 | Level 1 | Level 2 |
| IL-6 | 6.79 | 6.38 | 5.31 | 5.09 | 6.26 | 5.90 | 6.16 | 5.73 | 5.70 | 4.82 |
| IL-8 | 7.00 | 6.68 | 5.06 | 4.31 | 6.22 | 5.41 | 6.65 | 5.54 | 5.15 | 4.91 |
| IL-10 | 6.39 | 5.00 | 5.55 | 4.96 | 6.83 | 6.09 | 5.92 | 5.35 | 6.00 | 5.17 |
| IFN-α | 5.70 | 5.55 | 5.63 | 5.28 | 6.34 | 6.22 | 4.73 | 3.83 | 6.99 | 6.01 |
| IFN-γ | 6.86 | 5.44 | 5.92 | 5.21 | 6.06 | 5.34 | 5.67 | 4.92 | 7.11 | 6.30 |
| IL-12p70 | 5.19 | 4.91 | 5.47 | 4.95 | 6.72 | 5.78 | 5.69 | 5.16 | 6.14 | 5.61 |
| IL-17 | 5.88 | 5.32 | 5.16 | 4.71 | 6.92 | 5.84 | 5.35 | 5.11 | 6.67 | 5.85 |
| IL-1β | 6.69 | 6.54 | 6.18 | 5.40 | 5.57 | 5.09 | 5.45 | 4.41 | 7.08 | 6.40 |
| IL-2 | 6.79 | 6.49 | 5.52 | 5.10 | 6.38 | 5.83 | 6.59 | 5.84 | 6.07 | 5.37 |
| IL-4 | 7.48 | 6.66 | 6.12 | 5.58 | 6.82 | 6.17 | 5.90 | 5.50 | 6.14 | 5.54 |
| IL-5 | 6.13 | 5.77 | 5.37 | 4.53 | 5.97 | 5.45 | 5.83 | 5.18 | 5.11 | 4.68 |
| TNF-α | 5.58 | 5.25 | 5.28 | 4.43 | 6.08 | 5.60 | 5.18 | 4.03 | 6.08 | 5.12 |

CVs: coefficient of variations; IL-6: interleukin-6; IL-8: interleukin-8; IL-10: interleukin-10; IFN-α: interferon-α; IFN-γ: interferon-γ; IL-12p70: interleukin-12p70; IL-17: interleukin-17; IL-1β: interleukin-1β; IL-2: interleukin-12; IL-4: interleukin-4;IL-5: interleukin-5; TNF-α: tumor necrosis factor-α.

**Supplementary Table 2. Cytokine bias across five laboratories under two quality control levels (%)**

| Cytokines | Bias of Lab A | | Bias of Lab B | | Bias of Lab C | | Bias of Lab D | | Bias of Lab E | |
| --- | --- | --- | --- | --- | --- | --- | --- | --- | --- | --- |
|  | Level 1 | Level 2 | Level 1 | Level 2 | Level 1 | Level 2 | Level 1 | Level 2 | Level 1 | Level 2 |
| IL-6 | 2.29 | 0.84 | 0.64 | 2.73 | 2.41 | 1.15 | 1.52 | 1.45 | 0.75 | 1.60 |
| IL-8 | 2.23 | 0.42 | 0.22 | 0.66 | 0.68 | 0.05 | 2.93 | 1.40 | 0.19 | 1.22 |
| IL-10 | 1.54 | 0.98 | 2.91 | 0.36 | 0.52 | 0.03 | 0.10 | 0.60 | 1.99 | 1.26 |
| IFN-α | 0.56 | 0.37 | 0.07 | 0.85 | 1.06 | 0.12 | 1.24 | 1.58 | 0.45 | 1.94 |
| IFN-γ | 2.23 | 1.51 | 1.68 | 0.39 | 2.03 | 0.33 | 1.17 | 0.84 | 0.32 | 0.04 |
| IL-12p70 | 0.37 | 0.91 | 0.59 | 1.25 | 0.46 | 0.91 | 0.99 | 1.57 | 1.68 | 2.82 |
| IL-17 | 1.17 | 2.71 | 1.98 | 1.18 | 0.82 | 0.24 | 0.70 | 0.11 | 0.71 | 1.21 |
| IL-1β | 1.88 | 0.61 | 0.93 | 0.22 | 2.29 | 0.35 | 1.05 | 0.10 | 0.53 | 0.38 |
| IL-2 | 2.59 | 0.17 | 0.36 | 0.01 | 0.25 | 1.67 | 0.21 | 0.73 | 2.19 | 0.76 |
| IL-4 | 2.95 | 0.46 | 0.41 | 1.43 | 1.29 | 0.38 | 1.09 | 0.26 | 0.15 | 1.09 |
| IL-5 | 0.72 | 1.06 | 1.27 | 0.88 | 0.02 | 1.50 | 0.20 | 0.94 | 0.37 | 0.74 |
| TNF-α | 0.13 | 1.55 | 0.25 | 2.26 | 0.68 | 0.04 | 0.51 | 0.99 | 0.81 | 0.25 |

IL-6: interleukin-6; IL-8: interleukin-8; IL-10: interleukin-10; IFN-α: interferon-α; IFN-γ: interferon-γ; IL-12p70: interleukin-12p70; IL-17: interleukin-17; IL-1β: interleukin-1β; IL-2: interleukin-12; IL-4: interleukin-4;IL-5: interleukin-5; TNF-α: tumor necrosis factor-α.
